# Supplementary material for: Protective Effect of Flavonoids against Methylglyoxal-Induced Oxidative Stress in PC-12 Neuroblastoma Cells and Its Structure–Activity Relationships
Source: Molecules. 2022 Nov 12;27(22):7804. doi: 10.3390/molecules27227804 (PMC9693184; doi:10.3390/molecules27227804)
Supplement: Supplementary file 1 [file molecules-27-07804-s001.zip › molecules-2004557-supplementary.pdf]

Supplementary Materials

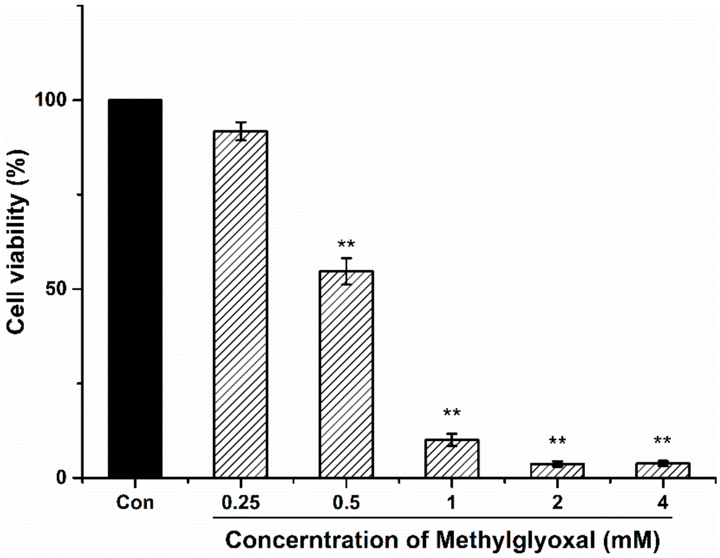

Figure S1. Cell viability of PC-12 cells treated by MG. * p<0.05, ** p<0.01 compared with the control.

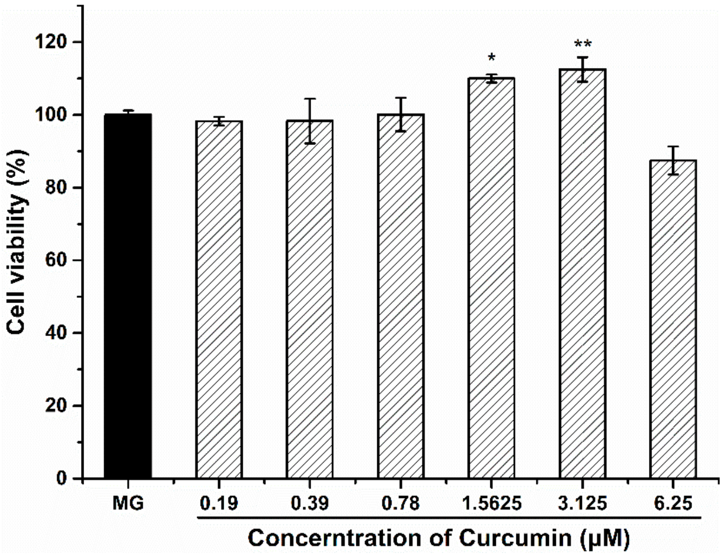

Figure S2. Cell viability of MG-induced PC-12 cells treated by curcumin. * p<0.05, ** p<0.01 compared with the MG group.

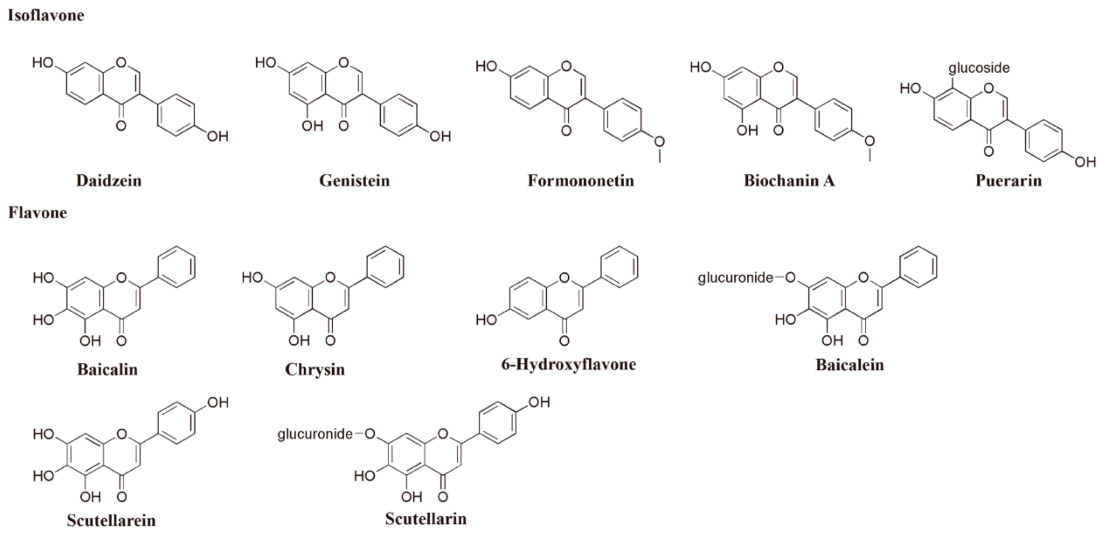

Figure S3. Other flavonoids tested in protective activities on MG-induced PC12 cells.

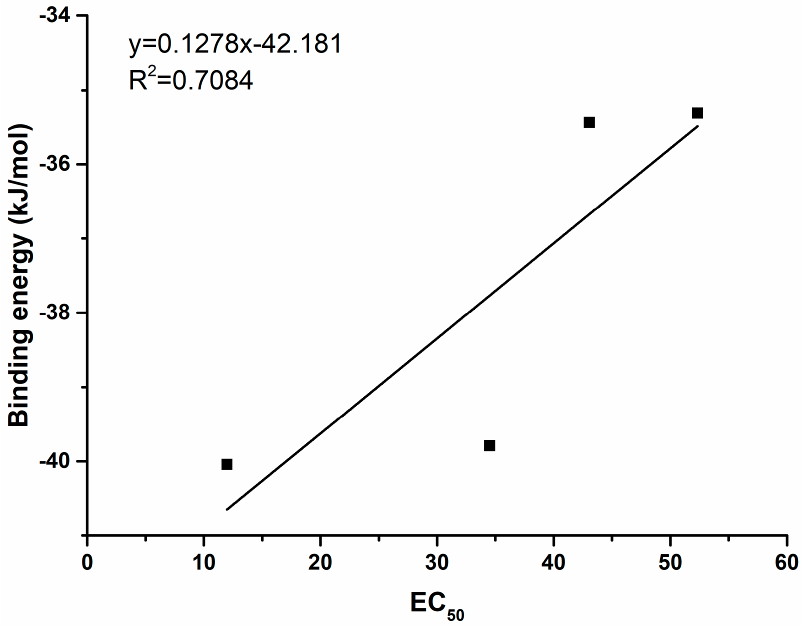

Figure S4. The linear correlation between binding energy of main flavan 3-ols (EC, EGC, ECG, EGCG) and the EC_{50} for MG-induced PC-12 cells.
